# Does heavy metal exposure affect the condition of Whitethroat (*Sylvia communis*) nestlings?

**DOI:** 10.1007/s11356-017-1064-1

**Published:** 2017-12-30

**Authors:** Katarzyna Turzańska-Pietras, Justyna Chachulska, Ludmiła Polechońska, Marta Borowiec

**Affiliations:** 1 Museum of Natural History, University of Wrocław, ul. Sienkiewicza 21, 50-335 Wrocław, Poland; 2 Department of Nature Conservation, Faculty of Biological Sciences, University of Zielona Góra, ul. prof. Z. Szafrana St. 1, 65-516 Zielona Góra, Poland; 3 Department of Ecology, Biogeochemistry and Environmental Protection, University of Wrocław, ul. Kanonia 6/8, 50-328 Wrocław, Poland

**Keywords:** Industrial pollution, Passerine nestlings, Biomonitoring, Bird excreta, Bird condition, Trace elements

## Abstract

Anthropogenic pollution results in high concentrations of heavy metals in the environment. Due to their persistence and a high potential for bioaccumulation, metals are a real threat for birds breeding in industrial areas. The aim of the present study has been to explore the contents of heavy metals (arsenic As, cadmium Cd, chromium Cr, copper Cu, iron Fe, nickel Ni, lead Pb and zinc Zn) in the excreta of Whitethroat (*Sylvia communis*) nestlings living in polluted environment and to investigate the relationship between these contents and the nestlings’ condition. Excrement samples contained all the studied elements. The contents of arsenic, cadmium, copper and zinc in the excreta of nestlings from nests located close to a slag dump were several times higher than in the soil near the dump, which suggested accumulation in food consumed by the birds. Condition parameters (body mass and haemoglobin concentration) were not related to heavy metal concentrations in the nestlings’ excreta, except of Zn. It is possible that Whitethroats are able to detoxicate heavy metals to a certain extent. Detailed, multi-element analysis of the environment, food and bird tissues or excreta should be performed to explore relations between different chemicals and bird condition.

## Introduction

Human-induced pollution caused by industrial processes is a common global problem. High concentrations of heavy metals, along with their persistence as well as a high potential for bioaccumulation and, for some elements, biomagnification, make them a real threat to various organisms and ecosystems (Berglund et al. 2011; Markert et al. 2003). Metals can affect birds in many ways, e.g. reducing their reproductive success and compromising growth and condition (Eeva and Lehikoinen 1996; Eeva et al. 2012). Studies on the influence of heavy metals on the condition of wild bird nestlings are scarce. Mainly a narrow group of passerine species: Great tit (*Parus major*), Blue tit (*Cyanistes caeruleus*) and Pied flycatcher (*Ficedula hypoleuca*), have been studied in this aspect so far (Janssens et al. 2003; Rainio et al. 2013; Berglund et al. 2014; Sánchez-Virosta et al. 2015).

Birds are known to use several ways to eliminate toxic metals from their body—through excrements, deposition in the uropygial gland and salt gland or excretion into growing feathers or eggshells (Dauwe et al. 2000; Costa et al. 2013). Therefore, metal contents can be assessed in birds without killing the individuals to examine their tissues. The amount of metals in feathers cleaned from external contamination is directly related to their contents in blood in the time of feather growth, while excrements reflect metal concentration in food recently consumed by the bird (Costa et al. 2013). Heavy metal contents in excreta correspond to food chain exposure to metal pollution. Investigating contents in faeces is a non-invasive and useful way to monitor the presence and concentrations of metals in birds’ food and habitat (Dauwe et al. 2000, 2004; Costa et al. 2013). Passerine nestlings’ excreta should accurately reflect current local pollution as they are the result of parents’ foraging activity in a limited area and are produced in a clearly defined time (Dauwe et al. 2000).

There are no studies concerning the effects of metal pollution on *Sylvia* warblers, although some of them inhabit and breed in ruderal, heavily polluted environments, e.g. suburban areas (Bednorz et al. 2000; Tomiałojć and Stawarczyk 2003). *Sylvia* warbler nestlings are mainly insectivorous, for example the diet of Whitethroat (*Sylvia communis*) nestlings consists of Lepidoptera (38–39%), Coleoptera (17%), Araneae (13–14%), Diptera (11–12%), Hymenoptera (7–9%) and Hemiptera (3–8%) (Moreby and Stoate 2001). Invertebrates, i.a. ground-dwelling spiders, are known to accumulate high heavy metal concentrations (Jung and Lee 2012) and some studies indicate that insects are a link in the transport of metals along food chains (Dauwe et al. 2004). Hence, due to their diet, *Sylvia* warbler nestlings may suffer from the bioaccumulation of toxins (Alleva et al. 2006; Abbasi et al. 2015).

The aim of this study was to explore the contents of eight heavy metals (arsenic As, cadmium Cd, chromium Cr, copper Cu, iron Fe, nickel Ni, lead Pb and zinc Zn) in the excreta of Whitethroat nestlings living in polluted environment and to investigate the relationship between these contents and the nestlings’ condition.

## Material and methods

### Study area

**Fig. 1:**
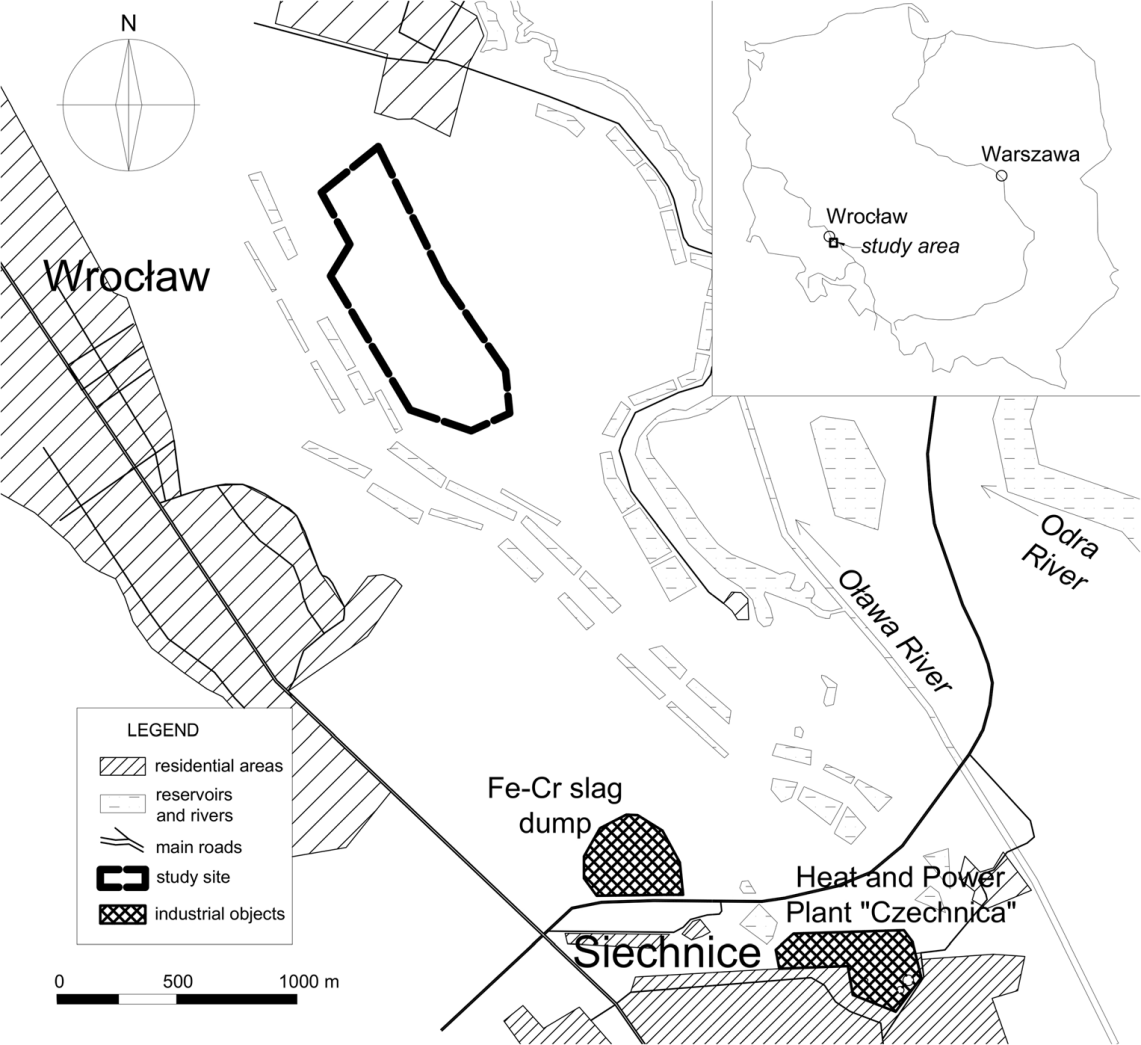
Map of the study area and position of the Fe-Cr slag dump, heat and power plant and the study site (dashed line) where samples were collected

In 2015 and 2016, we studied a Whitethroat population inhabiting the south part of water infiltration fields located to the south-east of Wrocław (Lower Silesia, SW Poland) (51° 03′41.8 ″ N, 17° 07′ 25.6″ E) (Fig. 1). The study area (about 54 ha) was covered by semi-natural, mown meadows and dense hawthorn (*Crataegus* sp.) and willow (*Salix* sp.) shrubs. The area was protected from human access because the infiltration fields were the source of drinking water for the Wrocław agglomeration. The region was considered polluted due to the vicinity of the heat and power plant “Czechnica” located in Siechnice and a slag dump of a ferrochrome steel plant that had been closed down in 1995. Trace metals (e.g. Cr, Zn, Cu, Fe, Mn, Cd), gases (sulphur dioxide SO_2_, nitrogen dioxide NO_2_, carbon dioxide CO_2_, carbon monoxide CO) and benzo[a]pyrene are known common pollutants in the vicinity of heat and power plant as well as steel plant. In particular, in the 1980s and 1990s, emission levels from the ferrochrome steel plant in Siechnice were about 220 t a^−1^ of Cr; 22 t a^−1^ of Fe and 46 t a^−1^ of dust containing As, Cd, Cu, Mn, Ni and Pb (Biłyk and Kowal 1993; Meinhardt et al. 2015). According to Szpadt (1987), Cr emissions from the plant were about 220 t a^−1^ in the last years of operation, the deposition onto water infiltration fields was 0.3–6.92 t km^−2^ and the range of environmental contamination was 10 km. In 2010, the heat and power plant “Czechnica” released 1440 t SO_2_, 2390 t NO_x_ and 111 t PM_10_ (Myllyvirta et al. 2013). Monitoring studies showed that dust emissions from the dump led to significant soil enrichment in Cr after closing the steel plant. In addition, the soil contents of other trace metals, Cd, Cu, Pb, Zn, were higher than the geochemical background (Table 1), suggesting the existence of pollution sources other than the slag dump (Biłyk and Kowal 1993; Karczewska and Bortniak 2008; Karczewska and Kabała 2010; Meinhardt et al. 2015). On the other hand, these contaminants may also come from traffic emissions from busy roads as well as fertilisers and fungicides used in the surrounding agricultural fields (Kabata-Pendias 2010). High content of trace elements (Co, Cu, Fe, Ni, Pb and Zn) was noted in aquatic macrophytes from reservoirs located in water infiltration fields (Samecka-Cymerman and Kempers 1994; Polechońska and Samecka-Cymerman 2016).

**Table 1:** Metal contents (μg/g d.w.) found in the soils in the study area and geochemical background values

Element	Content (μg/g d.w.)	Reference	Geochemical background in Poland (Kabata-Pendias and Pendias 1999)
As	4.3–9.04*	Meinhardt et al. (2015)	2–13
Cd	1.7–2.5*	Biłyk and Kowal (1993)	0.05–0.2
	0.250–0.426*	Meinhardt et al. (2015)	
Cr	45 ± 390**	Biłyk and Kowal (1993)	15–740
	45–210*	Karczewska and Bortniak (2008)	
	30.4–118*	Meinhardt et al. (2015)	
Cu	1.2 ± 4.4**	Biłyk and Kowal (1993)	5–19
	11–36*	Karczewska and Bortniak (2008)	
	9.5–22.1*	Meinhardt et al. (2015)	
Mn	200 ± 500**	Biłyk and Kowal (1993)	240–570
Ni	30 ± 57	Biłyk and Kowal (1993)	5–23
	7.46–19.5	Meinhardt et al. (2015)	
Pb	32 ± 108	Biłyk and Kowal (1993)	13–25
	21–84	Karczewska and Bortniak (2008)	
	19.3–32.6	Meinhardt et al. (2015)	
Zn	44 ± 210	Biłyk and Kowal (1993)	35–80
	64–387	Karczewska and Bortniak (2008)	
	38.1–148	Meinhardt et al. (2015)	

*Range

**Mean ± SD

The studied nests of the Whitethroat were situated in the part of the water infiltration fields that was considered to be within the range of contamination of the post-metallurgic dump (Biłyk and Kowal 1993): 1.735 to 2.655 km from the slag dump and 2.885 to 3.865 km from the power and heat plant “Czechnica” (Fig. 1.).

### Field methods

We collected the samples from mid-April to July, during breeding seasons of 2015 and 2016. We visited research area every day to localise singing males and search for nests. We marked the nestlings individually using numbered metal rings, and measured them when they were 6–7 days old. We weighed the nestlings using an electronic scale (to the nearest 0.01 g) and measured the concentration of haemoglobin using a portable haemoglobinometer (HemoCue Hb 201+ Analyser, Sweden). We took the blood samples (10–20 μL) by puncture from the wing vein using a sterile needle and a Pasteur pipette (Arctander 1988). Blood sampling was approved by the II Local Ethics Committee in Wrocław, Poland, under number 40/2014. If defecation occurred during handling, we collected the excreta immediately in clean labelled ziplock bags. We kept the samples in the freezer, until laboratory analysis. We recorded locations of the nests by a handheld Garmin GPS device and used Quantum GIS 2.14.1. to measure the distance between the nests and the slag dump, as well as the power and heat plant. After two seasons, we collected complete data (excreta, blood and body mass) from 29 breeding nests with 2–6 nestlings each (116 nestlings in total).

### Heavy metal analysis

Each piece of excreta was dried at 50 °C to a constant weight, weighted and grounded with an agate mortar and pestle. Subsequently, whole samples were digested with nitric acid (2 mL; 65%, analytical grade) in Teflon bombs with a MARS 5 microwave system (CEM Corporation, USA). The digests were diluted with deionised water to a total volume of 10 mL. As, Cd, Cr, Cu, Fe, Ni and Pb contents in the samples were determined using AAS with electrothermal atomisation and Zn with flame atomisation (AVANTA PM Atomic Absorption Spectrometer, GBC Scientific Equipment, Australia). All elements were assayed against Sigma Chemical Co. (Poznań, Poland) standards and blanks containing the same matrix as the samples. Element contents in excreta were calculated on a dry weight basis. The accuracy of the methods used in the laboratory procedures and determination of element contents was assessed against Certified Reference Materials: Peach Leaves (SRM 1547, National Institute of Standards and Technology, Gaithersburg, Maryland) and INCT-OBTL-5 (Oriental Basma Tobacco Leaves, Institute of Nuclear Chemistry and Technology, Warsaw, Poland). Recovery rates were found to be 98 ± 4 (percent ± standard deviation).

### Statistical analysis

After data exploration (Shapiro-Wilk *W* test for the normality of data and Bartlett’s test for the homogeneity of variances), non-parametric tests were selected for further analysis. Differences between nests in respect of metal contents in nestlings’ excreta, body mass and concentration of haemoglobin were checked by Kruskal-Wallis ANOVA. Further analyses were performed on means per nest. It is a common method used in bird toxicological studies (Janssens et al. 2003; Dauwe et al. 2004) and was suitable for our results because nests differed significantly in respect of all metal contents in their excreta, except for Cr and Pb, as well as in respect of body mass and Hb level (Kruskal-Wallis ANOVA, *p* < 0.05). Differences between samples collected in 2015 and 2016 in respect of metal contents in excreta and the condition parameters of nestlings (body mass, haemoglobin concentration) were checked by Mann-Whitney *U* test (*n* = 29). Results of the test showed no significant differences, and we decided to merge the 2 years in further analyses. In order to check the relationship between condition parameters and trace metal contents and to assess the influence of industrial emissions on the condition and mean trace metal contents of nestlings’ excreta, we analysed the relationship between heavy metal contents, body mass and Hb level and distance from the slag dump using Spearman correlation (*n* = 29).

To check the relationship between two condition parameters: body mass and haemoglobin concentration, Spearman’s correlation coefficient was used. In this analysis, every nestling was treated as a replicate (*n* = 116).

Multivariate adaptive regression splines (MARSplines) was used to find important factors affecting nestling’s condition and then as a regression model to describe the relationship between nestling’s condition and the important variables. MARSplines technique is a form of regression analysis introduced by Friedman in 1991. The model is a generalisation of techniques used for solving regression- and classification-type problems, in order to predict the value of a set of dependent or outcome variables from a set of independent or predictor variables. MARSplines algorithm operates as multiple piecewise linear regression, so it may show relations that are usually not found by simple regression models. The nonlinearity of a model is approximated through the use of separate slopes in each interval of the predictor variable range. The underlying function consists of a set of adaptive piecewise linear regressions (termed basis functions), that are directly determined from the data, the slope of which changes at points called knots. The model is non-parametric and does not require any a priori assumptions about the relationship between the dependent and independent variables (Nisbet et al. 2009; Mateo et al. 2010). In the present study, nestling’s body mass was used as dependent variable and trace metal concentrations in excreta were tested as the predictor variables. For each dependent variable, we ran few models using different parameters, e.g. maximum number of basic functions, interactions (to allow or prevent interactions between basic functions) and penalty for each knot added to the model to keep low complexity values (Mateo et al. 2010). In the end, the best model was chosen based on the generalized cross-validation (GCV) error value. In the final model, the penalty was set at 1 and the threshold was 0.0005, degree of interactions was 2, and pruning was applied. The GCV error was 0.813.

Statistical confidence was set at *p* = 0.05. Calculations were carried out using Statistica 12 (StatSoft Inc., 2016).

## Results and discussion

### Heavy metals in excreta

The excreta of the Whitethroat nestlings contained all the measured heavy metals (Table 2).

**Table 2:** Metal contents (μg/g d.w.) in the excreta, body mass (g) and haemoglobin concentration (g/L) of Whitethroat nestlings from study area and results of analysis of correlation (r_s_—Spearman correlation coefficient)

	Median	Min	Max	AD	r_s_ (distance to the slag dump)	r_s_ (body mass)	r_s_ (Hb concentration)
As (μg/g d.w.)	35.3	0.23	234	46.7	− 0.36	0.10	− 0.20
Cd (μg/g d.w.)	9.92	1.88	19.8	3.28	− 0.27	0.35	0.10
Cr (μg/g d.w.)	7.14	1.07	33.6	7.44	0.14	0.34	− 0.01
Cu (μg/g d.w.)	55.7	27.5	226	40.7	− 0.39*	0.19	− 0.09
Fe (μg/g d.w.)	1439	55.4	11,003	1820	0.01	0.28	0.03
Ni (μg/g d.w.)	6.41	1.57	27.0	5.57	0.18	0.22	− 0.17
Pb (μg/g d.w.)	4.76	1.45	43.6	6.39	0.17	0.26	0.16
Zn (μg/g d.w.)	704	108	1944	344	− 0.45*	0.39*	0.06
Body mass (g)	12.2	9.82	13.5	0.77	− 0.16	–	–
Hb (g/l)	113.6	95.8	142	8.23	− 0.05	–	–

*AD* average deviation

*Statistically significant correlations (*p* < 0.05)

The excreta had on average 3.9 times higher as concentration than the mean concentration in the soil of the meadows very close to the slag dump, measured by Meinhardt et al. (2014, 2015) in 2013 and 2014. On average, Cd, Cu and Zn concentrations were 23, 2.5 and 4.8 times as high, respectively, in the excreta as in the soil. These results indicate that metals were accumulated in food consumed by the nestlings. We found weak but significant negative correlation between Cu and Zn contents and distance from the slag dump (Table 2) indicating a decrease of contamination with growing distance to the pollutant. Dauwe et al. (2004) showed that the excreta of Great tit nestlings from nests located in the immediate vicinity of a non-ferrous smelter (0–0.35 km) had higher contents of some of the studied heavy metals (Ag, As, Cu, Hg and Zn) than the ones from sites situated at a distance from the source of pollution (0.4–0.6, 2.5 and 4 km).

Heavy metal contents noted in nestlings’ excreta were generally similar to results of studies performed on other passerine species in polluted areas. Nevertheless, probably due to the different sources of pollution, some values differed considerably (both between our and other authors’ results and among different studies on passerines). Excrements of Great tit nestlings living in the vicinity of metallurgic plants in Flanders and Belgium contained more As, Cd, Cu, Fe, Ni and Pb than in the present study (Dauwe et al. 2000; Janssens et al. 2003). The excreta of Great tit nestlings from area affected by a Cu/Ni smelter in Finland and polluted areas in Poland, in turn, showed lower contents of As, Cd, Ni, Pb and Zn than Whitethroats’ nestlings in our study (Nyholm et al. 1995; Berglund et al. 2011). However, comparisons of the effects of heavy metals in excreta or tissues on various bird species are very difficult, due to interspecies differences in ecology (especially diet) as well as ability to tolerate and detoxicate heavy metals (Koivula and Eeva 2010). Even closely related species like Great tits and Blue tits may vary significantly in their metal levels, which may be related to differences in their detoxification capacity or antioxidant defence, such as levels of antioxidant enzyme activity, which have been shown to differ between those species (Rainio et al. 2013). Belskii et al. (1995) showed that heavy metal contents in Pied flycatchers’ tissues and faeces were 1.4–3 times as high as in the Great tits inhabiting the same area. Berglund et al. (2011) also reported higher element contents in Pied flycatchers than Great tits. These observations were confirmed and explained by Belskii and Belskaya (2013) who studied contaminant exposure in two passerines as well as the contribution of different food components to bird metal intake. They concluded that bird species may vary in metal intake in the same habitat because of differences in proportions of invertebrate preys as well as differences in pollutant contents in the insects. What’s more, environmental conditions of the study site and source of pollution also play an important role, as elements interact with one another and the composition and proportion of certain elements may affect their impact on birds (Eeva and Lehikoinen 2004; Koivula and Eeva 2010).

### Heavy metals and nestlings’ condition

**Fig. 2:**
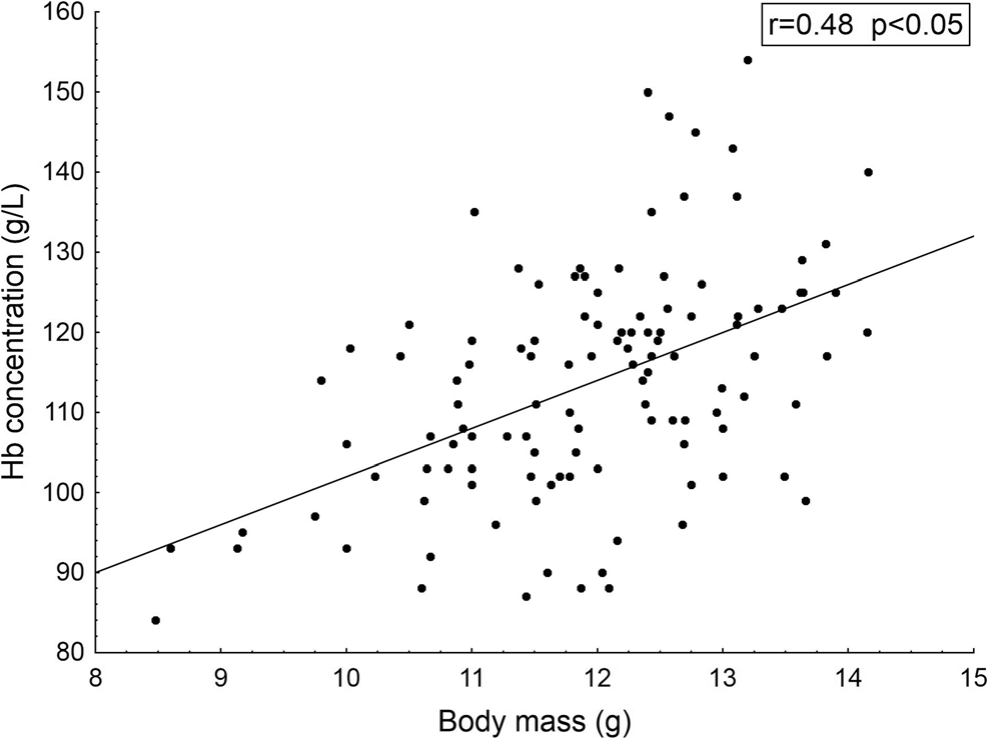
Relation between the haemoglobin concentration (g/L) in the blood of Whitethroat nestlings and their body mass (g) (*n* = 116). In the box, results of correlation analysis are presented: r—Spearman’s correlation coefficient; p—probability level

Median body mass of Whitethroat nestlings was 12.2 g (AD = 0.77, *n* = 29) and the median haemoglobin level was 113.6 g/L (AD = 8.23, *n* = 29) (Table 2). Body mass and haemoglobin concentration were significantly correlated (Fig. 2). Nestling mass depends on the quantity and quality of food provided by parents and it is broadly used as an index of nestling condition. Prefledging mass has been proved to predict survival to recruitment age in many bird species (Schwagmeyer and Mock 2008). Haemoglobin is responsible for oxygen transport in blood. Its concentration is one of the physiological indices of condition, which strongly depends on food supply (Kaliński et al. 2009). It may also reflect other aspects of a bird’s health, such as the blood parasite load (Słomczyński et al. 2006).

Low haemoglobin levels are associated with anaemia (Minias 2015) that may be caused by intoxication e.g. if birds are exposed to high levels of lead (Haig et al. 2014; Ferreyra et al. 2015). However, the haemoglobin concentrations in blood of the studied nestlings were higher than in a related species, Reed warbler *Acrocephalus scirpaceus* (89 g/L) from a non-polluted area in Lower Silesia, Milicz Ponds nature reserve, Sztwiertnia (2013). The average Hb concentrations in the present study were slightly lower than Hb concentration of healthy and well-developed Great Tits nestlings (126.39 ± 3.56 g/L) (Bańbura et al. 2011). Nyholm et al. (1995) gives 115 g/L as the lower limit of the normal Hb range in the Pied flycatcher at fledging (about 17 days old). According to Minias (2015), Hb concentration in nestlings’ blood increases and shows a peak just before fledging, while our birds were only 6–7 days old; Whitethroats usually fledge at the age of 10–12 days (Cramp and Brooks 1992). What’s more, we did not observe any death of the nestlings at the time of sampling that could be caused by metal toxicity, nor abnormalities in nestling development, reported in other species (Eeva and Lehikoinen 1996; Janssens et al. 2003). Most nestlings were very active during handling and, despite their young age, some of them jumped out of the nest while we approached, showing no symptoms of anaemia.

**Fig. 3:**
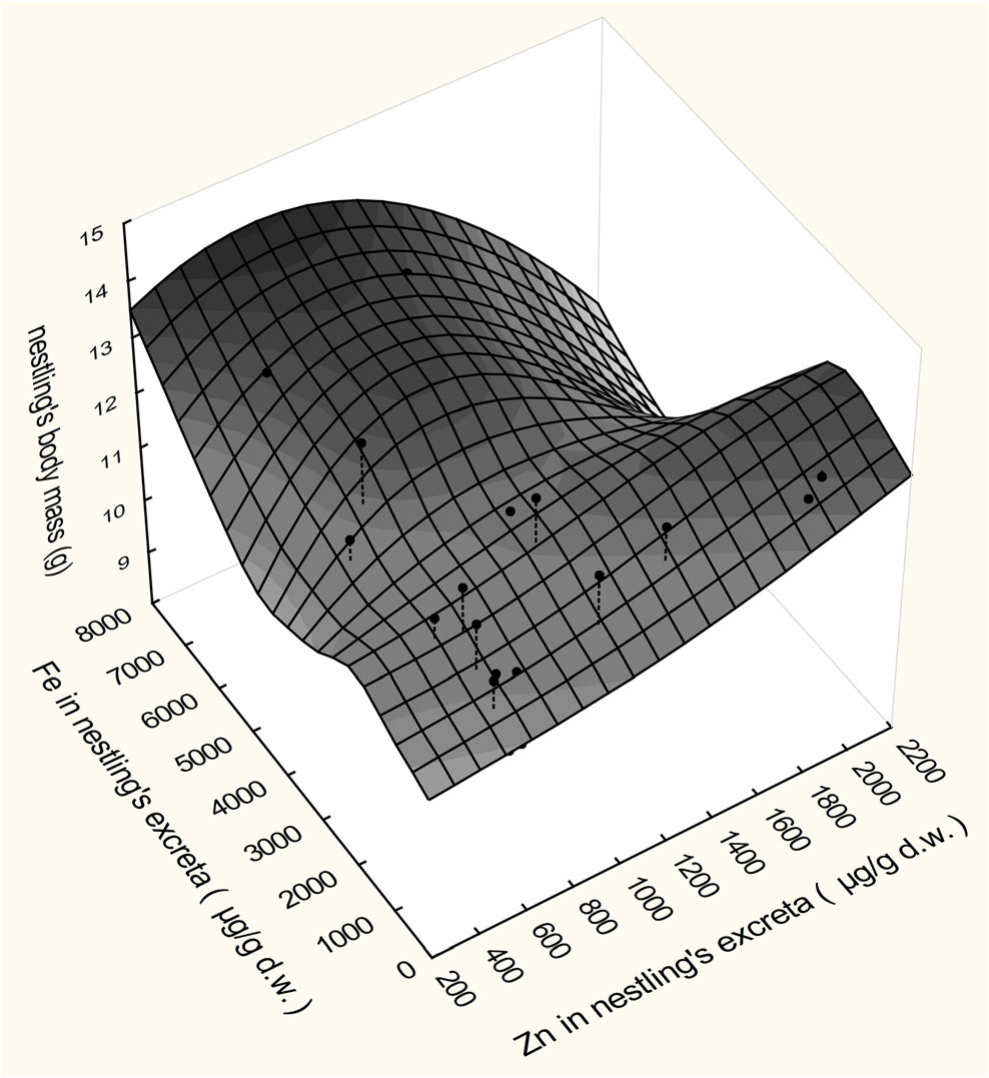
Multivariate adaptive regression splines (MARSplines) model of the relation between the body mass of Whitethroat nestlings and the content of Zn and Fe in their excreta (μg/g d.w.) (*n* = 29). Dots represent the actual values of parameters and the surface is a model regression surface. The equation of the regression: body mass = 1.12014564795804e + 001 + 2.06261682720149e-004*max(0; Fe-9.64650000000000e + 001) + 7.73563673027386e-004*max(0; Zn-2.97400000000000e + 002)

Metal contents in the nestlings’ excrements in the studied Whitethroat population generally did not correspond to their condition measured by body mass and haemoglobin concentration. Zn content only showed a weak but significant linear correlation with nestling body mass (Table 2). Also, the MARSplines model showed that Zn and Fe contents in excreta were important to predict the body mass of nestlings (Fig. 3). The model indicated that both low and high content of metals may adversely affect the body mass of birds; however, the relationship is complicated.

In previous studies, a clearly negative impact of heavy metal pollution on breeding success and nestling condition was shown. Janssens et al. (2003) showed that exposure of Great tit nestlings to high concentrations of heavy metals (Ag, As, Cd, Hg and Pb) resulted in reduction of their condition, including body mass. In the other research, it was proved that high mortality and growth abnormalities of Pied flycatcher nestlings as well as low breeding success and poor growth of young Great tits were inversely correlated to the distance form a copper smelter complex in SW Finland (Eeva and Lehikoinen 1996). Nyholm et al. (1995) showed that some Great tit nestlings in metal contaminated areas in Poland had lower than normal Hb levels which might have caused high nestling mortality. Lack of such a clear correlation in our study may be explained by the lower pollution level than in some of these study sites. For example, Eeva and Lehikoinen (1996) studied an area with much higher Cu, Ni and Zn contents in soil (up to 650, 359 and 432 μg/g d.w., respectively) (Fritze et al. 1989, Hyvarinen et al. 1993) and about 3.3 higher emission of SOx. On the other hand, it is possible that Whitethroats are able to detoxicate the metals to a certain extent into a less harmful form. Birds use various molecular mechanisms that enable them to tolerate poisonous compounds (Costantini 2008). For example, many different antioxidants (e.g. glutathione, vitamins, carotenoids) protect them against oxidative stress induced by trace metals (Koivula and Eeva 2010). Detailed investigation of this process in Whitethroats would be extremely valuable.

It is also possible that differences in the distance from the pollution source, and therefore the variation of heavy metal contents in our study, were too small to detect the variability of nestlings’ condition in respect to the pollution level. In the urban study site like ours, it is difficult to anticipate the ways and directions of heavy metal transport in the environment, because of many sources of contamination: heat and power plant “Czechnica”, slag dump from the liquidated ferrochrome steel plant, traffic emissions from busy roads, fertilisers and fungicides used in agriculture nearby (Cheng et al. 2014; Karczewska and Bortniak 2008). Nevertheless, our results suggest using a greater pollution gradient for future studies on Whitethroat vulnerability to contamination. In further studies, the availability of and metal content in food should also be taken into consideration. It was shown that prey promotes transfer of toxins into higher-level organisms and that this accumulation varies with metal presence in food and prey characteristics (Adair 2002; Belskii and Belskaya 2013).

The lack of reference values for condition parameters and heavy metal contents in excreta of the Whitethroats inhabiting non-polluted areas makes our results difficult to interpret. It is likely that this species is able to tolerate the metal concentrations encountered in the study site. However, it is also possible, that some more subtle effects on the nestlings’ condition parameters that we did not measure, e.g. haematocrit level, oxidative stress or telomere length, might have occurred (Koivula and Eeva 2010; Stauffer et al. 2017). We consider it unlikely that Whitethroats are able to detoxicate efficiently enough to overcome all the negative effects of heavy metal pollution, while metal toxicity in birds has been shown on organismal as well as physiological, cellular and genetic level (Koivula and Eeva 2010).

## Conclusions

Our results showed that in general heavy metal contents in the Whitethroat nestlings’ excreta were not directly related to their body mass and haemoglobin concentration. Only Zn concentration showed weak positive correlation with nestling’s body mass. Bigger birds excreted more metal than small ones. Analyses indicated that there may exist some more subtle and complicated relationships; however, this aspect needs further investigation. The influence of heavy metals on Whitethroat nestlings’ condition is a complex problem, which, due to difficulties in finding multiple nests in one area, collecting a large sample of excreta and determining an adequate pollution gradient, requires further investigation. Highly detailed, multi-element analyses of the environment, food and bird tissues or excreta should be performed to give the complete view of relations between different chemicals and bird condition. We recommend further investigation of this matter, as studies on other passerine species show various possible negative effects of metal contamination on birds.
